# Three-year follow-up of a randomized controlled trial comparing concomitant and staged treatment of varicose veins following mechanochemical ablation of the great saphenous vein

**DOI:** 10.1016/j.jvsv.2025.102255

**Published:** 2025-05-05

**Authors:** Tasnuva Rahman, Katariina Noronen, Sari Vähäaho, Ivika Heinola, Maarit Venermo, Karoliina Halmesmäki

**Affiliations:** a Department of Vascular Surgery, Helsinki University Hospital and University of Helsinki, Helsinki, Finland; b Department of Vascular Surgery, Päijät-Häme Central Hospital, Lahti, Finland

**Keywords:** Mechanochemical ablation, Randomized controlled trial, Varicose veins, Vascular diseases, Venous insufficiency

## Abstract

**Objective:**

Mechanochemical ablation is a feasible endovenous nonthermal, nontumescent treatment method for saphenous vein insufficiency. Nevertheless, the ideal approach to managing varicose veins following intervention of the saphenous trunk remains ambiguous. Treatment of varicose veins can be administered either simultaneously or in a staged manner. The aim of this 3-year follow-up study was to present the midterm outcomes of a randomized controlled trial, comparing concomitant and staged treatment of tributaries.

**Methods:**

Venous outpatient clinic patients with unilateral Clinical, Etiological, Anatomical, Pathophysiological (CEAP) C2-4 venous disease were enrolled in a randomized controlled trial during 2016 to 2017 at Helsinki University Hospital. After eligibility assessment of 1149 patients, 85 met the inclusion criteria: age of 20 to 70 years, ultrasound-verified refluxing above-knee great saphenous vein with a diameter of 5 to 10 mm, written consent from patients, and not having deep venous reflux, peripheral artery disease, pregnancy, lymphoedema, body mass index >40 kg/m^2^, allergy to the sclerosant, a history of deep vein thrombosis, or any form of coagulopathy. Participants were randomized, in a 1:1 ratio, to receive either staged tributary treatment with foam sclerotherapy at 3 months, if required (Group 1), or concomitant phlebectomies (Group 2), adjunct to mechanochemical ablation of the great saphenous trunk. All patients were invited to attend a 3-year follow-up, during which the initially treated leg was assessed with duplex ultrasound. The primary outcome was reintervention rate during follow-up. Secondary outcomes comprised presence of above-knee great saphenous vein reflux, patient satisfaction, status of the great saphenous vein, number of varicose veins, and symptoms at follow-up.

**Results:**

During follow-up, 11.4% (n = 5/44) (95% confidence interval [CI], 0.02-0.21) in Group 1 and 4.9% (n = 2/41) (95% CI, −0.02 to 0.11) in Group 2 was in need of additional treatment (Group 1 vs Group 2, odds ratio [OR], 2.5; 95% CI, 0.46-13.67; *P* = .435). The treatment groups did not elicit statistically significant variances in above-knee great saphenous vein reflux (*P* = .603), disease-specific and health-related quality of life (*P* = .238 and *P* = .255, respectively), status of the great saphenous vein (*P* = .112), or symptoms. However, noninferiority analysis suggests the staged approach to be inferior to the concomitant approach. Furthermore, Group 1 exhibited more varicosities at 3 years compared with Group 2, but this did not cause differences in the extent of symptoms or overall patient satisfaction.

**Conclusions:**

Staged treatment of tributaries in C2-4 venous disease provides acceptable midterm outcomes compared with simultaneous treatment. However, its potential inferiority should be taken into consideration when prioritizing durable treatment outcomes in the long term.

**Article Highlights**

• **Type of Research:** Three-year follow-up of a single-center prospective randomized controlled study
• **Key Findings:** At 3 years, reintervention rate and quality of life were similar between staged tributary treatment (44 patients) and concomitant tributary treatment (41 patients) adjunct to mechanochemical ablation of the great saphenous vein. However, noninferiority analysis indicated that the staged approach might be inferior to the concomitant approach.
• **Take Home Message:** Staged treatment of tributaries in C2-4 venous disease provides acceptable midterm outcomes compared with simultaneous treatment. However, its potential inferiority should be taken into consideration when prioritizing durable treatment outcomes in the long term.

Chronic venous disease (CVD) is a prevalent condition, affecting up to 40% of the adult population in Western countries.^1^ One notable manifestation of superficial venous insufficiency is the development of varicose veins, with studies indicating that approximately 30% of patients exhibiting great saphenous vein (GSV) incompetence also present with at least one tributary requiring treatment.^2^ The current gold standard for treatment involves endovenous laser ablation (EVLA) of the GSV trunk, accompanied by phlebectomies or foam sclerotherapy for side branches.^2^ Nevertheless, mechanochemical ablation (MOCA) has garnered attention as a feasible non-thermal, non-tumescent (NTNT) technique.^3^ Randomized controlled trials (RCTs) comparing MOCA and EVLA have shown MOCA to be both safe and efficacious at least in the short term,^4^ with guidelines suggesting MOCA to be a viable option for GSV ablation when a NTNT method is preferred.^2^

When side branches require interventions, treatment of the incompetent saphenous trunk and its tributaries can be performed either simultaneously or as a staged procedure.^5^ Guidelines emphasize that decisions regarding concomitant tributary treatment should be made through a shared decision-making process between the clinician and the patient, weighing the advantages and potential risks.^2^ To support this, RCTs providing definitive evidence on the optimal timing of side branch interventions along with the patient populations necessitating concomitant tributary management, both of which still remain uncertain, are needed.^3^ Although existing RCTs suggest that treating tributaries simultaneously may reduce the need for future reinterventions,^6^ there is also concern that treating tributaries during the same session could lead to overtreatment.^7^ Moreover, regression of varicose veins following saphenous trunk ablation alone has been observed in short-term studies, though the long-term effects of this phenomenon remain unexplored.^8^^,^^9^

In an effort to determine whether concomitant tributary treatment adjunct to MOCA of the saphenous trunk is necessary in patients with uncomplicated varicose veins, an RCT was conducted during 2016 to 2017, with the 1-year outcomes having previously been published.^3^ In the results, only MOCA of the main trunk has been shown to suffice for patients with Clinical, Etiological, Anatomical, Pathophysiological (CEAP) clinical class C2-C4 varicosities in the short term.^3^ However, the importance of longer follow-up was highlighted to draw more precise conclusions.^3^ Therefore, the aim of this study is to present midterm follow-up results of our trial and to assess the need for reinterventions and overall patient satisfaction at 3 years.

## Methods

In summary, we conducted a single center non-inferiority RCT during 2016 to 2017 in the Department of Vascular Surgery of Helsinki and Uusimaa Hospital District and in the University of Helsinki, Finland (registration number NCT03340246 at clinicaltrials.gov).^3^ This study has been given approval by The Ethics Committee of Helsinki University Hospital.^3^

All patients received ablation of the GSV using MOCA. Prior to the operation, tributary treatment was randomized using block randomization with sealed envelopes in a ratio of 1:1 to receive either no treatment (Group 1; n = 44) or concomitant phlebectomies (Group 2; n = 41). In Group 1, tributaries were treated with ultrasound-guided foam sclerotherapy (UGFS) at a 3-month follow-up visit, if required. Neither the surgeon nor the patient was blinded. The patient’s sex was confirmed using electronic health records of the patients.

### Procedures

All procedures were performed under ultrasound guidance in the outpatient department of the venous clinic of Helsinki University Hospital. In MOCA of the GSV, the ClariVein catheter (Vascular Insights) was employed, with 1.5% liquid sodium tetradecyl sulphate used as sclerosant.

Phlebectomies were done under local tumescent anesthesia (450 mL Ringer and 50 mL 1% lidocain cum adrenalin). One to nine skin incisions of 1 to 3 mm were made and covered with surgical tape afterwards.

UGFS of side branches was performed with sclerosant foam (1:3 sclerosant-to-air ratio) prepared using 1% polidocanol (Aetoxysclerol; Kreussler).

The patients wore a class 2 compression stocking from the procedure for the first 48 hours continuously and during the daytime for the next 5 days. Ibuprofen or paracetamol was prescribed upon necessity, the paracetamol with or without codeine. Wound care and aftercare were performed.

### Assessments

The primary outcome of this study was the need for additional treatments during 3-year follow-up. Secondary outcome measures included presence of above-knee GSV reflux (reflux >0.5 s), patient satisfaction, GSV status (occluded/partially recanalized/open, with presence of at least 5 cm patent and compressible GSV defined as partial recanalization), number of residual or recurrent varicose veins, and symptoms.

The need for reinterventions was determined based on standardized criteria outlined in current clinical practice guidelines.^5^ Patients with CEAP C4-6 venous disease underwent additional treatment whenever it was technically feasible. For those with CEAP C2-3 disease, further procedures were performed only when they experienced highly troublesome symptoms and significant reflux on duplex ultrasound of the treated leg.^5^

Patient satisfaction was determined by disease-specific quality of life (DSQoL), health-related quality of life (HRQoL), the patient’s subjective health state, and the patient’s perspective of the amount of help from the staff concerning patient communication at the 3-year visit. Patient communication was defined as the exchange of information between the hospital staff and the patient, encompassing explaining diagnoses, discussing treatment options, providing instructions, answering questions, and ensuring that the patient understands their treatment plan. DSQoL was measured using the Aberdeen Varicose Vein Questionnaire (AVVQ), a validated instrument used to assess the impact of varicose veins on the patient’s QoL.^10^ AVVQ scores range from 0 to 100, with higher scores indicating worse QoL.^10^ HRQoL was measured using a 15-dimentional, self-administered instrument called the 15D Questionnaire.^11^ 15D scores range from 0 to 1, with 0 indicating death and 1 perfect health.^11^

Noninferiority of staged tributary treatment (Group 1) compared with concomitant management of tributaries (Group 2) was assessed based on noninferiority analysis using DSQoL and HRQoL and the presence of statistically significant between-group differences in the primary and secondary outcome measures.

### Statistical analysis

The data were tested for normality by means of histograms and Kolmogorov-Smirnov and Shapiro-Wilks testing. Continuous normally distributed data were analyzed using the *t* test or the one-way analysis of variance and continuous non-normally distributed data through the utilization of the Mann-Whitney *U* test or the Kruskal-Wallis test. Categorical data were analyzed using the χ^2^ test, and Fisher’s exact test was applied when expected cell counts were less than five. Logistic regression was used to calculate odds ratios (ORs) and their 95% confidence intervals (CIs). A subgroup analysis was performed to investigate whether the interventional technique of side branch treatment within Group 1 (phlebectomy vs UGFS of varicosities) had a significant influence on the primary and secondary outcomes of this study. Specifically, patients in Group 1 were divided into two subgroups: Group A and Group B. Group A consisted of patients who did not receive UGFS of side branches at 3 months, and Group B included patients who underwent UGFS of side branches at the 3-month follow-up. In the subgroup analysis, Group C consisted of the patients who underwent concomitant phlebectomy as part of the primary intervention. Noninferiority analysis was done using mean AVVQ scores and mean 15D scores at 3 years, with the noninferiority margin being set at 5%. The *P*-value < .05 was regarded as statistically significant. Statistical analysis was performed using SPSS for Windows and MacOSX version 28.0.0.1 (14) (IBM).

## Results

**Fig 1:**
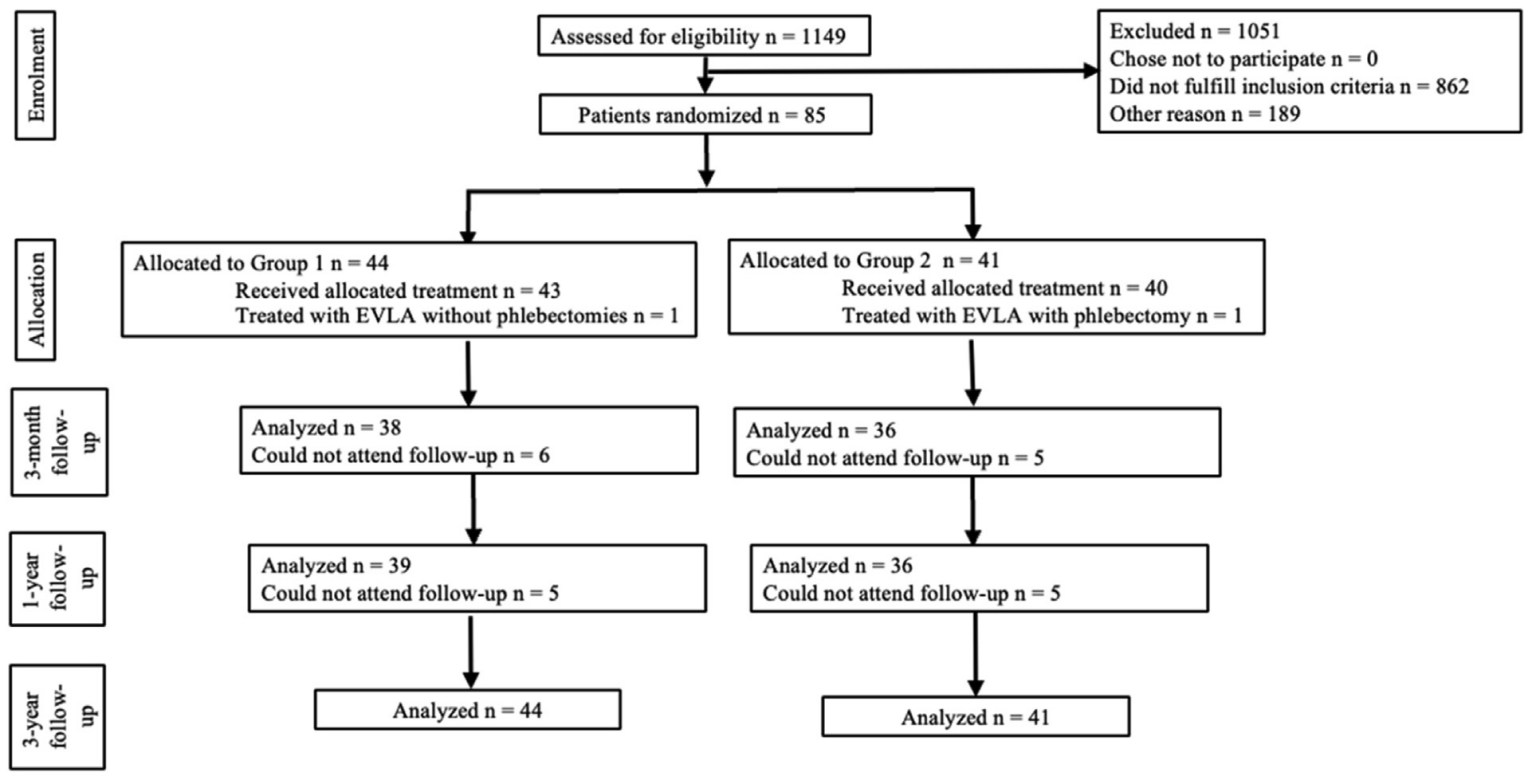
Study flow chart. *EVLA*, Endovenous laser ablation.

All 85 patients treated during 2016 to 2017 were invited to attend a midterm follow-up visit 3 years after the primary intervention. All the patients (100%) attended the 3-year follow-up (Fig 1). In Group 1, 54.5% (n = 24/44) answered the AVVQ and 65.9% (n = 29/44) the 15D questionnaire at 3 years; the respective number of responses in Group 2 was 70.7% (n = 29/41) and 73.2% (n = 30/41) patients (*P* = .188 for AVVQ and *P* = .624 for 15D). The overall response rate was 62.4% for AVVQ and 69.4% for the 15D questionnaire. The baseline attributes of the patients did not differ in statistical terms, except for the mean age being lower in Group 1 compared with Group 2.^3^ This did not affect analyses or the outcomes due to strict inclusion and exclusion criteria of this study and statistical similarity in the rest of the baseline characteristics between the treatment groups.^3^

At baseline, 97.7% (n = 43/44) patients in Group 1 and 92.7% (n = 38/41) patients in Group 2 presented with one to three side branches. Three to five side branches were exhibited by 2.3% (n = 1/44) patients in Group 1 and 7.3% (n = 3/41) patients in Group 2 at the baseline visit. No patient in either group demonstrated more than five side branches prior to the primary intervention. The groups did not elicit a statistically significant variance in the number of side branches at baseline (*P* = .349).

### Primary outcome

By the 3-year visit, 11.4% (n = 5/44) (95% CI, 0.02-0.21) in Group 1 and 4.9% (n = 2/41) (95% CI, −0.02 to 0.11) in Group 2 required additional treatment (Group 1 vs Group 2: OR, 2.5; 95% CI, 0.46-13.67; *P* = .435). Table I summarizes the supplementary interventions perfomed or scheduled for the patients post primary intervention.

**Table I:** Additional procedures performed during follow-up and scheduled at the 3-year visit for both treatment groups

	Group 1 (n = 44)	Group 2 (n = 41)
During follow-up		
Only EVLA of the GSV	1 (2.3)	0 (0.0)
UGFS for side branches	0 (0.0)	1 (2.4)
Scheduled at the 3-year follow-up		
EVLA of the GSV and the SSV	1 (2.3)	0 (0.0)
UGFS of the GSV	1 (2.3)	0 (0.0)
UGFS for side branches	1 (2.3)	0 (0.0)

*EVLA,* Endovenous laser ablation; *GSV,* great saphenous vein; *SSV,* short saphenous vein; *UGFS,* ultrasound-guided foam sclerotherapy.

Data are presented as number (%).

### Secondary outcomes

Key secondary outcomes are described in Table II.

**Table II:** Secondary outcomes in both treatment cohorts, including presence of reflux in the above-knee great saphenous vein (*GSV*), patients’ satisfaction with treatment, patients’ perception of their health, status of the GSV, varicose vein count and change, and symptoms

	Group 1 (n = 44)	Group 2 (n = 41)	*P*
Above-knee GSV reflux at 3 years	2 (4.5)	1 (2.4)	.603
Patients’ perception of own state of health at 3 years			.211
Good	38 (63.3)	35 (72.9)	
Moderate	2 (3.3)	5 (10.4)	
Low	4 (6.7)	1 (2.1)	
Patients’ perception of change in own state of health compared with baseline			.385
Better	34 (56.7)	33 (65.3)	
No change	8 (13.3)	8 (15.7)	
Worse	2 (3.3)	0 (0.0)	
Patients’ perception of amount of help from the staff concerning patient communication at 3-year visit			.554
Abundant amount of help	32 (54.2)	32 (61.0)	
Some help	7 (11.9)	7 (13.4)	
Insufficient help	5 (8.5)	2 (3.7)	
GSV status at 3 years			.075
Occluded	33 (58.9)	33 (66.0)	
Partially recanalized	6 (10.7)	8 (16.0)	
Open	5 (8.9)	0 (0.0)	
No. varicose veins at 3 years			< .001
None	19 (43.2)	36 (87.8)	
One or more	25 (56.8)	5 (12.2)	
Change in no. varicose veins compared with baseline			< .001
All or most varicose veins have disappeared	26 (59.1)	39 (95.1)	
All varicose veins still present	15 (34.1)	0 (0.0)	
New varicose veins	3 (6.8)	2 (4.9)	
Symptoms at 3 years			
Pain	7 (15.9)	9 (22.0)	.777
Edema	11 (25.0)	12 (29.3)	1.000

Data are presented as number (%).

### Quality of life outcomes

**Fig 2:**
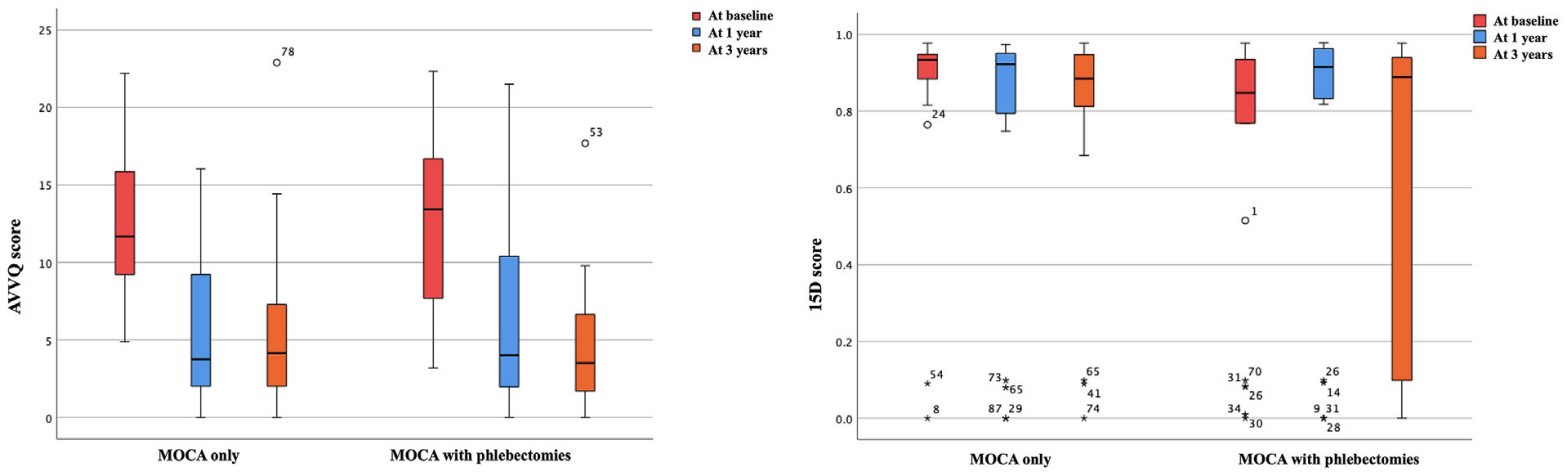
Aberdeen Varicose Vein Questionnaire (AVVQ) scores representing disease-specific quality of life (DSQoL) and 15D scores describing health-related quality of life (HRQoL) in each treatment group at baseline, 1 year, and 3 years. *Horizontal lines* within boxes represent medians, the *edges* of the boxes represent the interquartile ranges, the *whiskers* represent the range, and *open circles* illustrate outliers.

At 3 years, no statistically significant difference was observed in AVVQ scores between the groups (mean AVVQ score, 6.0 (standard deviation [SD], 5.7) in Group 1 and 4.8 (SD, 4.6) in Group 2; *P* = .587). Both groups demonstrated a similar and notable within-group improvement in AVVQ scores during follow-up (mean within-group change in AVVQ score during follow-up, −8.0 [SD, 7.0] in Group 1 and −8.8 [SD, 5.9] in Group 2; *P* = .247) (Fig 2).

At follow-up, 15D scores were statistically significantly higher in Group 1 compared with Group 2 (mean 15D score, 0.7727 [SD, 0.3054] and 0.6842 [SD, 0.3857] in Group 1 and Group 2, respectively; *P* = .044). 15D scores slightly improved in Group 1 and decreased in Group 2 during follow-up (mean within-group change in 15D score during follow-up, 0.0116 [SD, 0.4556] in Group 1 and −0.0984 [SD, 0.4911] in Group 2; *P* < .001) (Fig 2).

The difference in the mean AVVQ score is 2.68 (95% CI, −1.77 to 7.13) and the difference in the mean 15D score is 0.08 (95% CI, −0.11 to 0.28) between Group 1 and Group 2, respectively. The noninferiority margin is 0.46 for the AVVQ score and −0.03 for the 15D score. Because the lower bounds of the 95% CIs are less than the noninferiority margins for AVVQ and 15D scores, Group 1 does not fully meet the criteria for noninferiority compared with Group 2, neither in terms of DSQoL nor HRQoL.

### Results of the subgroup analysis

Subgroup analysis comparing Group 1 patients without staged UGFS of tributaries (Group A), Group 1 patients with staged UGFS of tributaries (Group B), and the patients who received concomitant phlebectomy during the primary intervention (Group C), showed no statistically significant differences in the following outcomes: need for additional treatments during follow-up (*P* = .549), status of the above-knee GSV (*P* = .131), above-knee GSV reflux (*P* = .652), patient satisfaction (*P* = .574), change in health state during follow-up (*P* = .151), DSQoL (*P* = .264), HRQoL (*P* = .309), pain (*P* = 1.000), and edema (*P* = .918). The groups elicited a statistically significant variance in subjective health state (*P* = .022), with Group B demonstrating worse subjective health compared with the other groups. Group A and Group C showed similar subjective health. A difference of statistical significance was also observed in the number of varicosities at 3 years (*P* < .001), with Group C exhibiting a lower number of varicosities compared with the other treatment cohorts. Group A and Group B demonstrated a comparable number of varicose veins.

## Discussion

In this trial, staged and concomitant tributary treatment in patients with C2-C4 venous disease resulted in comparable overall treatment outcomes 3 years post primary intervention. However, noninferiority analysis of DSQoL and HRQoL suggested the staged approach to be inferior to the combined approach. Furthermore, the latter group exhibited greater regression of varicose veins during follow-up (*P* < .001). Despite the potential inferiority of isolated GSV truncal treatment concerning QoL, no statistically significant variances in DSQoL or HRQoL were observed at 3 years. The more substantial regression of varicose veins did not translate into notable differences in symptom severity or overall patient satisfaction either. Thus, acceptable midterm treatment results can be achieved with MOCA along with the option of staged tributary treatment in management of GSV reflux with symptomatic varicose veins. These findings corroborate the 1-year outcomes of this trial; nevertheless, the subtle indications of the staged approach possibly being inferior to simultaneous tributary treatment should be taken into consideration when prioritizing durable treatment outcomes in the long term.^3^

Group 1 had a significantly higher prevalence of varicose veins at 3 years compared with Group 2 (*P* < .001) due to not receiving phlebectomies during the primary procedure. However, this difference lacked clinical significance. Group 2 did, however, exhibit a more pronounced reduction in varicosities during the follow-up period (*P* < .001). This observed reduction in varicose veins cannot be attributed to a higher rate of side branch treatments in Group 2 but instead may result from a decrease in venous pressure achieved through surgical excision of side branches.^12^ Phlebectomy mitigates high-pressure reflux, thereby reducing overall venous pressure and curtailing blood pooling in smaller tributaries, which facilitates venous remodeling and subsequent regression of smaller varicose branches over time.^13^ Moreover, inflammatory mediators that foster new varicosity formation are reduced post phlebectomy, potentially aiding in the regression of smaller varicosities.^14^

Previous research has indicated that GSV occlusion rates tend to decline over time when the primary intervention involves MOCA, possibly necessitating additional GSV treatments and impacting long-term cost-effectiveness.^15^ This aligns with the observed occlusion rates in this trial: 79.3% at 3 years compared with 89.7% at 1 year in Group 1, and 84.4% at 3 years vs 94.4% at the 1-year follow-up in Group 2. Given this midterm decrease in occlusion, venous reflux may recur in the long term, potentially causing persistence or resurgence of varicosities.^16^ New varicosities, although subclinical in presentation, emerged in both treatment groups over the course of follow-up. If the trend of GSV recanalization continues, these newly formed tributaries may progress to symptomatic varicose veins, potentially necessitating further interventions. This reflects the persistent nature of CVD, suggesting a possibility of need for supplemental tributary interventions alongside MOCA of the main trunk regardless of the timing of tributary treatment. Nonetheless, reflux in the above-knee GSV was minimal in both groups at 3 years, noted in only 4.5% of patients (n = 2/44) in Group 1 and 2.4% of patients (n=1/41) in Group 2, indicating midterm treatment success. Furthermore, in a 5-year follow-up study of an RCT comparing concomitant and staged tributary treatment by El-Sheikha et al, the concomitant approach resulted in more favorable clinical and QoL outcomes compared with staged treatment of tributaries.^17^ Therefore, a sustained follow-up is essential to confirm the observations made in our present study over an extended period.

Omitting side branch treatment may result in residual varicosities, potentially posing a cosmetic concern. Nevertheless, concurrent phlebectomy carries risks such as pigmentation changes and telangiectasia formation, which are avoided when treating only the GSV trunk.^18^ Only four patients across both treatment arms expressed significant concern over their leg appearance, a comparable number between the groups. Although cosmetic factors are not typically formal criteria for treatment in publicly funded health care, they can influence QoL and are relevant in patient-centered decision-making.^19^ With regard to other complications, a retrospective analysis of 954 limbs in 711 patients revealed significantly higher prevalence of pain in non-phlebectomy patients than those treated with EVLA and phlebectomy.^20^ Similarly, patients in Group 1 reported greater pain at 3 months in this trial, although no between-group differences were observed at 1 or 3 years.^3^ A study from 2021 also reported paresthesia as the most common complication associated with concomitant phlebectomy, although this was not observed in our study.^21^

The most recent clinical practice guidelines from the Society for Vascular Surgery and the American Venous Forum recommend addressing tributaries simultaneously with the ablation of the incompetent saphenous trunk.^2^ However, when anatomical or medical contraindications are present, a staged approach to managing tributaries is advised.^2^ The guidelines emphasize the importance of shared decision-making between patients and clinicians to determine the most appropriate timing for tributary treatment.^2^ The findings of this study, which indicate potential inferiority of the staged approach, underscore the importance of carefully considering patient preferences while prioritizing long-term durability to achieve the best possible outcomes.

QoL outcomes constitute a critical determinant of treatment success in C2-C4 class venous disease; hence, a limitation of this study was the somewhat lower-than-anticipated response rate of the QoL questionnaires (62.4% for the AVVQ and 69.4% for the 15D questionnaire). However, the treatment groups exhibited no statistically significant differences in the proportion of patients responding to the QoL questionnaires (*P* = .188 for the AVVQ and *P* = .624 for the 15D). With a limited number of responses to the QoL questionnaires and the relatively small size of our patient population, the likelihood of a Type II error is substantially heightened. This is particularly probable in the context of the AVVQ score; had the full cohort participated, the *P*-value in the between-group difference might have reached statistical significance.

Achieving satisfactory results in Group 2 with only one to nine incisions during the primary procedure suggests that most of these patients had mild C2 varicose disease with relatively small side branches, potentially limiting generalizability of the findings of this study to more severe cases. However, delayed side branch treatment is typically more appropriate for mild cases, whereas severe disease often necessitates immediate varicosity removal during the primary procedure. Thus, the study population, which predominantly consisted of patients with mild disease, better reflects the specific patient group we intended to investigate. Moreover, although the size of the tributaries undeniably affects the number of incisions, it is not the only determining factor—the anatomical location and tortuosity of the side branches, as well as the surgeon’s technique, could also play a role.

Another limitation of this study is that side branches were treated with phlebectomy during the primary procedure but with UGFS at the 3-month follow-up. We opted for UGFS over phlebectomy for the staged procedure as it does not require tumescent anesthesia, making it more practical for follow-up visits. We also anticipated that the residual tributaries would be small enough for effective treatment with UGFS. Also, UGFS avoided the need for a new sick leave, minimizing burden to patients and their employers. Although subjective health and varicosity counts differed among the groups in the subgroup analysis, the analysis demonstrated that overall mid-term treatment outcomes cannot be attributed to the use of a different treatment modality for tributary management during primary intervention and at the 3-month visit post primary procedure. Moreover, both phlebectomy and UGFS are well-established methods for managing tributaries.^2^ Also, initial varicose vein size did not likely differ between groups due to 1:1 randomization and similar baseline CEAP clinical class.^22^ However, the results of the subgroup analysis should be interpreted with caution due to the small overall sample size.

A further limitation is that reinterventions in this study were unrestricted and could involve any treatment method, potentially influencing mid-term outcomes through the efficacy of these subsequent procedures. Also, the term midterm follow-up is somewhat ambiguous, typically ranging from 2 to 5 years in studies reporting outcomes related to CVD.^2^^,^^5^^,^^23^^,^^24^ Midterm results can increase understanding of the trajectory of the disease, but further follow-up is needed due to risk of varicose recurrence or new symptoms leading to an underestimation of long-term complications in studies limited at midterm follow-up. In addition, the absence of blinding for patients or surgeons could have introduced bias in the assessment of trial outcomes. Also, variability in the expertise of the surgeons performing the procedures could result in inconsistency in treatment outcomes.

Even though MOCA has not established superiority over EVLA,^15^ the current gold standard for saphenous truncal treatment,^22^ MOCA was selected as the GSV ablation method due to scarce research existing on the optimal timing of tributary treatment adjunct to MOCA of the GSV and clinical guidelines recommending it as a viable NTNT option for saphenous trunk ablation.^22^ During the initiation of the present trial, VenaSeal was unavailable in Finland, and although RFA was an option, clinicians preferred EVLA due to its use of a 6F sheath for greater precision, compared with a 7F sheath in RFA. EVLA also offered a finer 1-cm measurement scale, whereas the shortest treatment tip with RFA was 3 cm, limiting accuracy. However, considering the uncertainties surrounding long-term occlusion rates associated with MOCA,^15^ the authors of this study strongly advocate for investigations assessing long-term outcomes of MOCA of the GSV and reiterate the importance of longer-term follow-up studies evaluating the treatment strategies compared in this research.

## Conclusions

Staged treatment of tributaries in C2-4 venous disease with GSV reflux yields acceptable midterm outcomes compared with simultaneous tributary treatment. However, potential inferiority of the staged approach should be taken into consideration when prioritizing durable outcomes in the long term.

## Author contributions

Conception and design: KH, MV

Analysis and interpretation: TR

Data collection: KN, SV, IH, MV, KH

Writing the article: TR

Critical revision of the article: TR, KN, SV, IH, MV, KH

Final approval of the article: TR, KN, SV, IH, MV, KH

Statistical analysis: TR

Obtained funding: Not applicable

Overall responsibility: TR

## Funding

This study was funded by 10.13039/100008376Helsinki University Hospital. T.R. received a 2000 euro salary for this work from Helsinki University Hospital. Helsinki University Hospital had no involvement in the study design or collection, analysis, interpretation of data, the manuscript writing process, and the creation of the manuscript. Helsinki University Hospital was not involved in the decision to submit the manuscript for publication.

## Disclosures

None.

## References

[1] Vähäaho S., Halmesmäki K., Albäck A., Saarinen E., Venermo M. (2018). Five-year follow-up of a randomized clinical trial comparing open surgery, foam sclerotherapy and endovenous laser ablation for great saphenous varicose veins. Br J Surg.

[2] Gloviczki P., Lawrence P.F., Wasan S.M. (2024). The 2023 Society for Vascular Surgery, American Venous Forum, and American Vein and Lymphatic Society clinical practice guidelines for the management of varicose veins of the lower extremities. Part II: Endorsed by the Society of Interventional Radiology and the Society for Vascular Medicine. J Vasc Surg Venous Lymphat Disord.

[3] Rahman T., Vähäaho S., Laukontaus S. (2023). Randomized controlled trial comparing immediate and delayed treatment of varicose veins following mechanochemical ablation. JVS Vasc Insights.

[4] Mohamed A.H., Leung C., Wallace T., Smith G., Carradice D., Chetter I. (2021). A randomized controlled trial of endovenous laser ablation versus mechanochemical ablation with ClariVein in the management of superficial venous incompetence (LAMA trial). Ann Surg.

[5] Boersma D., Kornmann V.N., van Eekeren R.R., van der Wijk J.P., de Maeseneer M.G., van den Bos R.R. (2020). Three-year results of a randomized controlled trial comparing mechanochemical ablation to radiofrequency ablation for the treatment of great saphenous vein incompetence. J Vasc Surg Venous Lymphat Disord.

[6] Lane T.R.A., Kelleher D., Shepherd A.C., Franklin I.J., Davies A.H., The AVULS Trial Investigators (2015). A multicentre randomised controlled trial comparing simultaneous versus delayed phlebectomy after endovenous laser ablation: the AVULS trial. Eur J Vasc Endovasc Surg.

[7] Lane T.R., Kelleher D., Shepherd A.C., Franklin I.J., Davies A.H. (2015). Ambulatory varicosity avulsion later or synchronized (AVULS): a randomized clinical trial. Ann Surg.

[8] Lurie F., Creton D., Asonitis N. (2003). A randomized trial of endovenous laser treatment for varicose veins. Phlebology.

[9] Rasmussen L.H., Lawaetz M., Bjoern L., Vennits B., Blemings A., Eklof B. (2006). Randomized clinical trial comparing endovenous laser ablation, radiofrequency ablation, foam sclerotherapy, and surgical stripping for great saphenous varicose veins. J Vasc Surg.

[10] Klem T.M., Sybrandy J.E., Wittens C.H., Essink Bot M.L. (2009). Reliability and validity of the Dutch translated aberdeen varicose vein questionnaire. Eur J Vasc Endovasc Surg.

[11] Sintonen H. (2001). The 15D instrument of health-related quality of life: properties and applications. Ann Med.

[12] Park Y., Kim Y.W., Park Y.J., Kim D.I. (2015). Postoperative hemodynamic changes after endovenous laser ablation and phlebectomy in varicose vein surgery. J Vasc Surg Venous Lymphat Disord.

[13] Pfisterer L., König G., Hecker M., Korff T. (2014). Pathogenesis of varicose veins - lessons from biomechanics. VASA.

[14] Gwozdzinski L., Pieniazek A., Gwozdzinski K. (2024). Factors influencing venous remodeling in the development of varicose veins of the lower limbs. Int J Mol Sci.

[15] Vähäaho S., Halmesmäki K., Mahmoud O., Albäck A., Noronen K., Venermo M. (2021). Three-year results of a randomized controlled trial comparing mechanochemical and thermal ablation in the treatment of insufficient great saphenous veins. J Vasc Surg Venous Lymphat Disord.

[16] Baccellieri D., Ardita V., Pannone A. (2024). Factors influencing recurrent varicose vein formation after radiofrequency thermal ablation for truncal reflux performed in two high-volume venous centers. J Vasc Surg Venous Lymphat Disord.

[17] El-Sheikha J., Nandhra S., Carradice D. (2014). Clinical outcomes and quality of life 5 years after a randomized trial of concomitant or sequential phlebectomy following endovenous laser ablation for varicose veins. Br J Surg.

[18] Goldman M.P. (2011).

[19] Hitchman L.H., Mohamed A., Smith G.E. (2023). Provision of NICE-recommended varicose vein treatment in the NHS. Br J Surg.

[20] Malik R., Lakhanpal S., Mavor A.I., Graham D., Eng A. (2020). Phlebectomy reduces postoperative pain following endovenous laser ablation: a retrospective cohort study of 954 limbs in 771 patients. Ann Vasc Surg.

[21] Brown C.S., Obi A.T., Cronenwett J.L., Kabnick L., Wakefield T.W., Osborne N.H. (2021). Outcomes after truncal ablation with or without concomitant phlebectomy for isolated symptomatic varicose veins (C2 disease). J Vasc Surg Venous Lymphat Disord.

[22] De Maeseneer M.G., Kakkos S.K., Aherne T. (2022). European Society for Vascular Surgery (ESVS) 2022 clinical practice guidelines on the management of chronic venous disease of the lower limbs. Eur J Vasc Endovasc Surg.

[23] Elalfy A., Elawdy M.M., Omar G.H., Abdelrahman M.A. (2019). Early and mid-term results of treatment of superficial venous insufficiency by endovenous laser ablation. Egypt J Hosp Med.

[24] Brittenden J., Cooper D., Dimitrova M. (2019). Five-year outcomes of a randomized trial of treatments for varicose veins. N Engl J Med.

